# The Interplay between Emotion and Cognition in Autism Spectrum Disorder: Implications for Developmental Theory

**DOI:** 10.3389/fnint.2012.00113

**Published:** 2012-12-04

**Authors:** Sebastian B. Gaigg

**Affiliations:** ^1^Department of Psychology, Autism Research Group, City University LondonLondon, UK

**Keywords:** autism, emotion, social-motivation, social brain, social cognition

## Abstract

Autism Spectrum Disorder (ASD) is a neurodevelopmental disorder that is clinically defined by abnormalities in reciprocal social and communicative behaviors and an inflexible adherence to routinised patterns of thought and behavior. Laboratory studies repeatedly demonstrate that autistic individuals experience difficulties in recognizing and understanding the emotional expressions of others and naturalistic observations show that they use such expressions infrequently and inappropriately to regulate social exchanges. Dominant theories attribute this facet of the ASD phenotype to abnormalities in a social brain network that mediates social-motivational and social-cognitive processes such as face processing, mental state understanding, and empathy. Such theories imply that only emotion related processes relevant to social cognition are compromised in ASD but accumulating evidence suggests that the disorder may be characterized by more widespread anomalies in the domain of emotions. In this review I summarize the relevant literature and argue that the social-emotional characteristics of ASD may be better understood in terms of a disruption in the domain-general interplay between emotion and cognition. More specifically I will suggest that ASD is the developmental consequence of early emerging anomalies in how emotional responses to the environment modulate a wide range of cognitive processes including those that are relevant to navigating the social world.

## Introduction

Ever since the behavioral syndrome now recognized under the rubric of the Autism Spectrum was first described more than six decades ago, it has been noted that atypicalities in reciprocal emotion related behaviors constitute a hallmark feature of its clinical presentation. Both Kanner (1943) and Asperger (1944) identified difficulties in this domain as a unifying characteristic amongst the cases they described, and to this day diagnostic instruments and screening tools consider anomalies in emotional reciprocity as a clinically significant indicator of the disorder (Schopler et al., 1980; Lord et al., 1989, 1994; Robins et al., 2001). For as long as disturbances in affective processes have been considered central to the clinical phenotype of *Autism Spectrum Disorder* (ASD), however, their significance in the development of the disorder has remained a matter of debate. Kanner (1943) and Asperger (1944) originally disagreed over whether they had identified a biologically determined disorder of interpersonal affect or a particular personality trait. During the 1950s and 1960s views diverged over the misguided concern of whether the primary caretakers might be to blame for their child’s emotional withdrawal or not, and most recently the dispute has turned to the question of whether early emerging impairments in social-cognitive processes are responsible (e.g., Baron-Cohen, 1995, 2005; Frith, 2003; Schultz, 2005), or whether Kanner’s (1943) original emphasis on innately specified interpersonal affective processes may have been correct after all (e.g., Hobson, 2002; Loveland, 2005; Chevallier et al., 2012). As diverse as the ideas have been over the decades, they have all shared the view that emotion related difficulties in ASD are to be understood with reference to atypicalities in social processes. In this review I will question this position and suggest instead that the social-emotional characteristics of the disorder are more fruitfully conceptualized in terms of domain-general anomalies in how the influences of emotions on cognition, that sometimes augment and at other times attenuate experiences of the world, organize what an infant learns about salient events in a complex and dynamically changing environment. In the context of the broader issues under consideration in this Special Topic, the study of ASD is therefore of considerable interest as it sheds light on a critical developmental function of the interplay between emotion and cognition.

The Autism Spectrum comprises a set of related pervasive developmental disorders (PDDs) that are all characterized by atypicalities in the domains of communication and socialization and by a restricted and repetitive pattern of interests and activities (Wing and Gould, 1979; American Psychiatric Association, 2000). The disorders subsumed under this spectrum include *Autistic Disorder*, *Asperger’s Disorder*, and PDD-*Not Otherwise Specified* (PDD-NOS), which together affect approximately 1% of the population and are around three to four times more common in males than females (Bertrand et al., 2001; Baird et al., 2006). All subtypes of ASD share a uniquely patterned cognitive profile that includes difficulties in understanding behaviour in terms of mental states, such as beliefs and desires (Baron-Cohen et al., 1985; Baron-Cohen, 1995; Frith, 2003), difficulties in deploying cognitive resources flexibly in order to plan and execute goal directed behaviors (Ozonoff et al., 1991a; Hill, 2004), a tendency to process information in a piecemeal and perceptually driven rather than holistic and conceptually driven fashion (Shah and Frith, 1993; Mottron and Burack, 2001; Frith, 2003; Mottron et al., 2006) and a characteristic profile of memory strengths and weaknesses that parallels that seen in neuropathologies of the frontal and/or medial-temporal lobes (Boucher and Bowler, 2008; Boucher et al., 2012). Since there is little evidence to support a nosological differentiation of subtypes of autistic pathologies (see Wing and Gould, 1979; Wing, 1993; Prior et al., 1998; Volkmar et al., 2004; Bowler, 2007 for discussion), the forthcoming edition of the Diagnostic and Statistical Manual of Mental Disorders 5^1^ will include only the single category of “ASD,” which will be the preferred term used throughout this review. I will describe individuals who have received a diagnosis of an ASD as *autistic individuals* rather than *individuals with autism* to reflect the preferred terminology of those who are on the spectrum (see Pellicano et al., 2011).

In relation to emotions, the view I adopt in this review is informed by several influential authorities in the field (e.g., James, 1890; Cannon, 1927; Schachter and Singer, 1962; Reisenzein, 1983; Lazarus, 1984; Zajonc, 1984; Ekman, 1992; Levenson, 1992; Damasio, 1994, 1999, 2003; LeDoux, 1996, 2002; Lane and Nadel, 2000; Russell, 2003; Lane, 2006; Barrett et al., 2007). I will use the words “*emotion(al)*” and “*affect(ive)*” in a theoretically neutral sense to describe the phenomena that accompany situations that tend to elicit approach and avoidance behaviors. The term “*arousal*” will denote automatic changes in physiological parameters such as heart rate, skin conductance, or pupil dilation that occur during (but are not specific to) emotional episodes. Although arousal is also often used to describe changes in brain activity (e.g., cortical arousal), I will restrict my discussion here to changes in peripheral nervous system activity that are, broadly speaking, open to conscious awareness. The word “*feeling*” will be used to describe the subjective experience that people report when they are asked about their emotional state. It does not comprise “feelings” of mere physical sensations (e.g., I feel cold) or homeostatic imbalances (e.g., I feel sick) although the two are closely related. The critical difference is that emotional feelings necessarily involve an *evaluation* (often termed appraisal) of arousing events whilst physical feelings are primarily conscious perceptions of the body’s internal milieu. The nature of the *evaluative* process in question in this context remains the matter of debate but most authors agree that it is intricately linked to self-awareness and consciousness on the one hand and self-preservation on the other (e.g., LeDoux, 2002; Damasio, 2003; Lane, 2006). More specifically, stimulus induced arousal is thought to shape (or elicit) perceptions of arousing objects or events in terms of their *value* (innately specified or learned) to the wellbeing (physical or psychological) of the Self, which modifies how the Self is expressed in consciousness (e.g., as feeling afraid). Finally, it is important to note that this review will not deal with the topic of mood. Although moods and emotions are closely related, only emotions are intentional in the philosophical sense of being about something and the focus of this paper is on how autistic individuals experience, understand, and relate to the objects and events in the world that emotions are about.

## The Social-Emotional Difficulties Associated with ASD

Although emotion related processes have been of interest in relation to ASD since Kanner (1943) first described the syndrome, work in this area has almost exclusively focused on the processes and behaviors that serve to regulate interpersonal conduct. Within this narrow focus, the literature is vast, covering more than 100 empirical papers and a broad theoretical literature. Doing justice to this work in the space available is difficult and I must therefore apologize to all authors whose views I have caricatured in the service of brevity. Fortunately, the three points I want to make in this section are relatively uncontroversial. The first is, that it is widely accepted that ASD is characterized by difficulties in multiple facets of interpersonal emotional communication. The second, that dominant explanations of these difficulties attribute them to anomalies in the development of broader reciprocal social competences. And the third, that these reciprocal social competences are mediated by a network of cortical and sub-cortical regions that collectively constitute what has often been called the “social brain.”

### The evidence

Amongst the earliest studies to systematically assess emotion related behaviors in ASD was a series of experiments by Hobson and colleagues who showed that autistic children were limited in understanding the emotional expressions of others. Such children experienced difficulties in matching emotional expressions across facial, vocal, and gestural modalities (Hobson, 1986a,b; Hobson et al., 1988a) and they appeared not to take notice of emotional expressions when asked to sort photographs of faces in whatever way they wished (Weeks and Hobson, 1987). When they were instructed to sort faces according to their expressions, autistic children were able to do so but through a more piecemeal perceptual processing style (Hobson et al., 1988b).

The pioneering work by Hobson and colleagues remains exemplary in terms of methodological rigor and ingenuity and it also remains representative of the observations of the 100 or so studies that followed. This last conclusion may come as a surprise because brief summaries of the relevant literature in empirical papers often give the impression that it is not entirely clear whether or not autistic individuals perceive and identify emotional expressions differently. Fortunately, a recent review of the facial emotion recognition literature in ASD by Harms et al. (2010) clarifies this issue. Following their systematic scrutiny of over 40 behavioral, 7 eye-tracking, and 22 brain imaging and electrophysiological studies up to 2010, Harms et al. (2010) conclude that, despite inconsistencies in behavioral observations, there is little doubt that autistic individuals extract emotions from faces differently than comparison groups. Tables 1 and 2 below are an attempt to complement Harms et al. (2010) by summarizing emotion perception studies in ASD across modes of expression. Table 1 provides a brief description of behavioral studies up to April 2012 with studies failing to demonstrate convincing group differences listed first. Table 2 summarizes functional brain based studies for the same period^2^. In line with Harms et al. (2010), the available evidence reveals inconsistencies in behavioral findings with approximately one in every four studies failing to demonstrate atypical emotion perception in ASD. These inconsistencies do not appear to be systematically related to a single methodological factor such as the nature of the experimental paradigm used, the type or number of emotions studied, or sample characteristics (e.g., age, ability level) including the procedures used to match ASD and comparison groups (e.g., VA vs. NVA). In combination these factors do account for some of the inconsistencies and certain co-morbidities also appear to play a role (e.g., Cook et al., in press). Importantly, however, brain based studies reveal atypical neural correlates of emotion perception relatively consistently in ASD, suggesting that not all behavioral paradigms are sensitive to qualitative differences in *how* autistic individuals extract emotional information from the face. Overall therefore, the conclusions by Harms et al. (2010) in relation to facial emotion recognition hold for emotion perception on the whole, and to the best of my knowledge, no authors have ever claimed that autistic individuals identify and understand the emotional expressions of others in an entirely typical fashion.

**Table 1:** **Studies examining emotion perception in ASD**.

Reference	Participant groups	*N*	Matching	Paradigm	Emotional expressions studied						
					Happy	Sad	Anger	Fear	Surprise	Disgust	Other
**STUDIES FAILING TO NOTE ASD SPECIFIC ATYPICALITIES**											
Ozonoff et al. (1990, Exp. 1)	ASD; TD	14; 14	VA	Sort static photographs of faces according to emotional expression	x	x	x				
Prior et al. (1990)	ASD; TD; DD	20; 20	VA	Match static schematic facial expressions with vocalizations, gestures, and contexts	x	x	x	x			
Loveland et al. (1997)	ASD; TD; DD	35; 23; 18	VA	Identify expressions from prosodic verbal and non-verbal vocal stimuli	x	x	x		x		
Serra et al. (1999)	PDD-NOS; TD	31; 31	CA; FSA	Explain how situational contexts influence a protagonist’s actual and displayed emotions	Not specified						
Buitelaar et al. (1999)	ASD; TD; DD	40; 20; 20	CA; FSA; VA; NVA	Match static facial expressions with one another and with situational contexts	x	x	x	x	x	x	x
Adolphs et al. (2001, Exp. 1)	ASD; TD; Amyg lesion	6; 28; 3	FSA	Discriminate static facial expressions of various intensities	x	x	x	x	x	x	
Adolphs et al. (2001, Exp. 2)	ASD; TD; Amyg lesion	7; 18; 8	Not specified	Identify static facial expressions	x	x	x	x	x	x	
Gepner et al. (2001)	ASD; TD	13; 13	≠CA; FSA	Identify facial expressions from static, smooth dynamic, or strobe dynamic displays (ASD performance differently modulated by experimental manipulations)	x	x			x	x	
Hillier and Allison (2002)	ASD; TD; DD	10; 20; 10	CA; VA; NVA	Influence of audience on judgments of embarrassment of a protagonist							x
Robel et al. (2004)	ASD; TD	20; 20	CA	Identify and match static facial expressions with one another	x	x		x	x		
Castelli (2005)	ASD; TD	20; 20	VA	Identify and match static facial expressions with one another	x	x	x	x	x	x	
Ashwin et al. (2006a)	ASD; TD	18; 18	CA; FSA	Visual search for static schematic facial expressions	x		x				
Begeer et al. (2006, Test 2)	ASD; TD	28; 32	CA	Select photographs of faces according to what person is most likely to offer a sweet or tell someone off	x		x				
Miyahara et al. (2007)	ASD; TD	20;20	CA; NVA	Identify expressions in static and dynamic real and cartoon faces (subtle group differences apparent when individual differences are explored)	x					x	
Wright et al. (2008)	ASD; TD	35; 35	CA; VA; NVA; FSA	Identify emotional expression in static faces or in richly contextual scenes (subtle group differences in relation to anger and happiness)	x		x	x	x	x	
Homer and Rutherford (2008)	ASD; TD	8; 12	CA; VA; NVA; FSA	Identify static facial expressions varying in intensity to determine category boundaries and match static expressions with one another after a delay	x	x	x	x	x	x	
Hubert et al. (2009)	ASD; TD	16; 16	CA	Identify dynamic facial expressions vs. identify the age (old/young) of the same stimuli (ASD group exhibited lower GSR responses)	x		x				
Krysko and Rutherford (2009)	ASD; TD	19; 19	CA; VA; NVA; FSA	Visual search for static facial expressions (subtle differences in terms of the effect of distracter numbers)	x		x				
Baker et al. (2010)	ASD; TD	19;19	CA	Identify emotions from prosodic vocalizations presented dichotically (one vocalization to each ear)	x	x	x				
Grossman et al. (2010)	ASD; TD	16;15	CA; VA; NVA	Identify emotions from prosodic vocalizations	x	x					
Williams and Happé (2010)	ASD; TD	21; 21	CA; VA; NVA	Define and describe experiences of target emotions and identify facial expressions of the same emotions in dynamic video-clips	x	x		x	x	x	x
Schwenck et al. (2011)	ASD; conduct disorder; TD	55; 70; 67	CA; FSA	Identify emotional expression as they emerge in dynamic face videos (subtle group differences in relation to sadness)	x	x	x	x		x	
Rosset et al. (2011)	ASD; TD	30; 30	CA	Visual search for static schematic facial expressions	x		x				
Chevallier et al. (2011)	ASD; TD	16–20; 16–20 across three Exp.	CA; VA	Identify (from two alternatives) the emotion or internal state expressed in vocalizations; ASD group performed quantitatively similar to TD but were slower to respond in Exp. 3 under high cognitive load	x	x	x	x	x	x	
Jones et al. (2011)	ASD; DD; TD	99; 26; 31	CA; VA; NVA; FSA	Identify static facial expressions and expressions from prosodic verbal and non-verbal vocal stimuli (subtle group differences in relation to surprise)	x	x	x	x	x	x	
Brennand et al. (2011)	ASD; TD	15; 15	CA; ≠VA	Identify expression from prosodic vocalizations (group effect marginally significant; *p* = 0.083)	x	x	x	x			
Tracy et al. (2011)	ASD; TD	29; 31	CA; VA; NVA; FSA	Determine whether briefly presented static faces express a target emotion	x	x	x	x	x	x	x
**STUDIES DEMONSTRATING EMOTION PERCEPTION DIFFICULTIES IN ASD**											
Hobson (1986a)	ASD; TD; DD	23; 38; 11	CA; VA; NVA	Match static schematic facial expressions with gestures, vocalizations, and situational contexts	x	x	x	x			
Hobson (1986b)	ASD; DD	13; 13	CA; NVA	Match schematic drawings of gestures with videos of vocalizations and static facial expressions	x	x	x	x			
Weeks and Hobson (1987)	ASD; DD	15; 15	CA; VA	Sort static face photographs varying on emotional and non-emotional dimensions according to preference	x	x					
Hobson et al. (1988a)	ASD; DD	21; 21	CA; VA	Match vocal expressions with static facial expressions	x	x	x	x	x	x	
Hobson et al. (1988b)	ASD; DD	17; 17	CA; VA	Sort whole or partial static face photographs according to expression and sort upright and inverted faces according to expression or identity	x	x	x	x			
Braverman et al. (1989)	ASD; TD	15; 15	NVA	Identify and match static facial expressions with one another (no difference when matching on VA)	x	x	x	x			
Macdonald et al. (1989)	ASD; TD	10; 10	CA; NVA	Identify emotion from situational contexts and vocal recordings	x	x	x	x			
Tantam et al. (1989)	ASD; DD	10; 10	CA; NVA	Identify upright and inverted static facial expressions (no differences on identifying mismatching facial expressions)	x	x	x	x	x	x	
Ozonoff et al. (1990, Exp. 2)	ASD; TD	14; 14	NVA	Match static facial expressions with one another, with vocalizations, and with situational contexts (no difference when matching on VA)	x	x	x				
Smalley and Asarnow (1990)	ASD; TD	9; 9	CA; NVA	Identify and match static facial expressions with one another	Not specified						
Ozonoff et al. (1991a)	ASD; DD	20; 20	CA; FSA; VA; NVA	Match static facial expressions with one another	x	x	x	x	x	x	x
Ozonoff et al. (1991b)	ASD; DD	23; 20	CA; FSA; VA; NVA	Match static facial expressions with one another	x	x	x	x	x	x	x
Capps et al. (1992)	ASD; TD	18; 14	CA; FSA; VA; NVA	Describe personal experiences of certain emotions (ASD group experienced difficulties describing pride and embarrassment)	x	x					x
Fein et al. (1992)	ASD; TD	15; 30	VA; NVA	Match static facial expressions with situational contexts	x	x	x	x			
Yirmiya et al. (1992)	ASD; TD	18; 14	CA; FSA; VA; NVA	Identify emotion experienced by protagonists in video segments and report own emotional reaction to it	x	x	x	x			x
Baron-Cohen et al. (1993)	ASD; TD; DD	15; 15; 12	CA; VA	Match schematic and photographed static facial expressions with one another	x	x			x		
Davis et al. (1994, Exp. 1)	ASD; TD; DD	20; 10; 10	CA; VA; NVA	Match static face photographs varying on emotional and non-emotional dimensions according to a sample		x	x		x		
Davis et al. (1994, Exp. 2)	ASD; TD; DD	19; 11; 20	CA; VA; NVA	Match static facial expressions with one another	x	x	x		x		
Bormann-Kischkel et al. (1995)	ASD; TD; DD	41; 41	CA; NVA	Identify static facial expressions	x	x	x	x	x	x	x
Loveland et al. (1995)	ASD; DD	28; 28	CA; ≠VA; NVA; FSA; gender	Match dynamic facial expressions with appropriate vocalizations that are either synchronous or not	x	x	x		x		
Buitelaar and van der Wees (1997)	ASD; DD/TD	40; 40	CA; VA; NVA; FSA; (TD ≠ CA and gender)	Match static facial expressions with one another and select static facial expressions to match with contextual scenes	x	x	x	x	x	x	x
Baron-Cohen et al. (1997, Exp. 3)	ASD; TD	16; 16	CA; FSA	Identify static facial expression from whole faces or only the eye-region (ASD group particularly worse on “complex” mental states)	x	x	x	x	x	x	x
Moore et al. (1997)	ASD; TD; DD	13; 13	CA; VA	Describe point-light displays of people enacting emotional and non-emotional behaviors	x	x	x	x	x		
Celani et al. (1999)	ASD; DD; TD	10; 10; 10	CA; VA	Match static facial expressions with one another and select preferred expression	x	x					
Dennis et al. (2000)	ASD; TD	8; 8	CA; VA	Identify the emotional expression of a story character’s actual feeling and the emotion that would be expressed for the purpose of deception	x	x					
Boucher et al. (2000)	ASD; DD; TD	19; 19; 19	CA; VA; NVA (TD ≠ CA)	Identify vocal expressions of emotions and match static facial expressions with vocalizations (ASD worse than TD but not worse than DD)	x	x	x	x	x	x	
Grossman et al. (2000)	ASD; TD	13; 13	CA; FSA; VA	Identify static facial expressions accompanied by no, congruent, or incongruent verbal labels	x	x	x	x	x		
Howard et al. (2000)	ASD; TD	9; 10	CA; VA;	Identify expressions from static faces	x	x	x	x	x	x	
Teunisse and de Gelder (2001)	ASD; TD	17; 48	Not specified	Identify and match static facial expressions with one another	x	x	x	x			
Pelphrey et al. (2002)	ASD; TD	5; 5	CA	Identify static facial expressions	x	x	x	x	x	x	
Bölte and Poustka (2003)	ASD; TD; schizophrenia	35; 22; 21	NVA	Identify static facial expressions	x	x	x	x	x	x	
Losh and Capps (2003)	ASD; TD	28; 22	CA; VA	Identify emotion experienced by protagonists in video segments and define emotions through verbal descriptions	x	x	x	x	x	x	x
Heerey et al. (2003)	ASD; TD	25; 21	CA; VA; FSA	Indentify static facial expressions	x	x	x	x	x	x	x
Gross (2004, Exp. 1)	ASD;DD	27; 81	CA; FSA	Identify static facial expressions of humans, orang-utans, and canines	x	x	x		x		
Gross (2004, Exp. 2)	ASD;DD	18; 30	CA; FSA	Identify static facial expressions from whole, top-half or bottom half of human, orang-utan, or canine faces	x	x	x		x		
Gross (2005)	ASD; DD	24; 59	CA; FSA	Match static facial expressions of humans or canines with one another	x	x	x				
Ashwin et al. (2006b)	ASD; TD	26; 26	CA; FSA; VA	Identify static facial expressions	x	x	x	x	x	x	
Begeer et al. (2006, Test 1)	ASD; TD	28; 31	CA	Match static face photographs varying on emotional and non-emotional dimensions according to preference	x		x				
Golan et al. (2006)	ASD; TD	21; 17	CA; FSA; VA; NVA	Label static facial expressions and vocalizations	x	x	x	x	x	x	x
Kamio et al. (2006)	ASD; TD	18; 18	CA; FSA	Rate likeability of Japanese ideographs preceded by subliminal or supraliminal static facial expressions	x			x			
Lindner and Rosén (2006)	ASD; TD	14; 16	CA; VA	Match static facial expressions with static or dynamic facial expressions and with vocalizations	x	x	x				
Dyck et al. (2006)	ASD; DD; TD	30; 24; 449	≠CA; VA; NVA	Ability to identify emotions from static faces and prosodic vocalizations correlates atypically highly with VA in ASD	x	x	x	x	x	x	
Peppé et al. (2007)	ASD; TD	31; 72	VA; ≠CA	Prosodic assessment battery including test of ability to discern “liking” and “disliking” from prosody							x
McCann et al. (2007)	ASD; TD	31; 72	VA; ≠CA	Prosodic assessment battery including test of ability to discern “liking” and “disliking” from prosody							x
Rutherford and McIntosh (2007)	ASD; TD	10; 10	CA; VA; NVA; FSA	Decide which of two schematic facial expression drawings varying in emotional intensity looks more like the expressions seen in real life	x	x	x	x	x	x	
Ashwin et al. (2007); task outside scanner	ASD; TD	13; 13	CA; FSA	Identify static facial expressions		x	x	x	x	x	
Hubert et al. (2007)	ASD; TD	19; 19	CA	Describe point-light displays of people enacting emotional and non-emotional behaviors	x	x	x	x	x		
Mazefsky and Oswald (2007)	AS; HFA compared to normative data	15; 14	CA; ≠VA; NVA; FSA	Identify static facial expressions and emotions from prosodic vocalizations. (HFA but not AS group compromised in comparison to normative data)	x	x	x	x			
Humphreys et al. (2007)	ASD; TD	20; 18	CA; VA; NVA; FSA	Identify static facial expressions that are blended in various proportions with one another (e.g., 20% fear – 80% surprise). (No differences when discriminating between blended emotions)	x	x	x	x	x	x	
Boraston et al. (2007)	ASD; TD	11; 11	CA; VA; NVA	Identify emotions in anthropomorphically animated shapes and in static facial expressions	x	x	x	x			
O’Connor (2007)	ASD; TD	18; 18	CA	Identify static facial expressions when presented alongside congruent or incongruent vocalizations (no differences on isolated modalities)	x	x	x				
Tardif et al. (2007)	ASD; TD	12; 24	≠CA; VA; NVA	Identify facial expressions in static images or dynamic videos varying in speed and that are or are not accompanied by concordant vocalizations	x	x			x	x	
Shamay-Tsoory (2008)	ASD; TD	18; 21	CA; years in education	Point out the target (out of four) of a character’s (represented by a schematic face) envy and gloating by using emotional expressions as cues							x
Dziobek et al. (2008)	ASD; TD	17; 18	CA; FSA	Identify emotion experienced by protagonist in photographic scene and report own emotional reaction to it	Not specified						
Rosset et al. (2008)	ASD; TD	20; 40	CA; FSA	Categorize emotional expressions of upright and inverted static cartoon and human faces	x	x	x				
Santos et al. (2008)	ASD; TD	21; 21	CA	Identify static expressions from hybrid superimposed high-pass and low-pass face images	x	x					
Wallace et al. (2008, Exp. 1)	ASD; TD	28; 26	CA; VA; NVA	Identify static facial expressions	x	x	x	x	x	x	
Wallace et al. (2008, Exp. 2)	ASD; TD	15; 15	CA; VA; NVA; FSA	Identify expressions in static face parts (eyes only, mouth only, eyes + nose, mouth + nose, whole face)			x	x	x	x	
Corden et al. (2008a)	ASD; TD	21; 21	CA; VA; NVA; FSA	Identify static facial expressions	x	x	x	x	x	x	
Clark et al. (2008)	ASD; DD; TD	15; 10; 11	≠CA; VA	Identify emotion in briefly presented static faces	x		x				
Kätsyri et al. (2008)	ASD; TD	20; 20	CA; NVA; FSA; ≠VA	Identify expressions from static and dynamic face images that vary in the amount of low spatial frequency information	x		x	x		x	
Grossman and Tager-Flusberg (2008)	ASD; TD	25; 25	CA; VA; NVA	Order static whole face or eye-region images taken from dynamic expression videos in the correct order	x	x	x	x		x	
Da Fonseca et al. (2009)	ASD; TD	19; 19	CA	Identify emotions from situational contexts	x	x	x	x			
Kuusikko et al. (2009)	ASD; TD	57; 33	≠CA; gender	Identify expressions from the eye-region of static faces	x	x	x	x	x	x	
Akechi et al. (2009)	ASD; TD	14; 14	CA; VA; NVA; FSA	Discriminate between angry and fearful static whole face or eyes only expressions when gaze is direct vs. averted			x	x			
Rump et al. (2009, Exp. 1)	ASD; TD	19; 18	CA; VA	Identify facial expressions of varying intensities portrayed in dynamic video-clips	x	x	x	x			
Rump et al. (2009, Exp. 2)	ASD;TD	71; 72	CA; VA; NVA; FSA	Identify facial expressions of varying intensities portrayed in dynamic video-clips (particularly older adults with ASD were compromised)	x	x	x	x			
Atkinson (2009)	ASD; TD	13; 18	CA; VA; NVA; FSA	Identify emotions and instrumental action from whole body dynamic dot-light or fully illuminated bodies	x	x	x	x		x	
Bal et al. (2010)	ASD; TD	17; 38	CA; VA; NVA; FSA	Identify dynamic facial expressions as quickly as possible	x	x	x	x	x	x	
Law Smith et al. (2010)	ASD; TD	21; 16	CA; VA; FSA	Identify dynamic facial expressions varying in intensity	x	x	x	x	x	x	
García-Villamisar et al. (2010)	ASD; DD	19; 28	CA; ≠FSA	Identify and match static facial expressions	x	x					
Philip et al. (2010)	ASD; TD	23; 23	CA (subgroups also on FSA)	Identify emotions from face, gesture, and prosodic vocal stimuli	x	x	x	x	x	x	
Evers et al. (2011, three experiments	ASD; TD	17–23; 17–23 accross Exp.	CA; FSA	Match static facial expressions with one another or with dynamic expressions across varying task demands (ASD worse than TD as demands increase)	x		x		x	x	
Krebs et al. (2011)	ASD; TD	24; 24	CA; FSA	Classify static faces either according to emotional expression or identity. (ASD group slower in a manner that indicates qualitative differences)	x	x					
Farran et al. (2011)	ASD; TD	20; 40	subgroups on either CA or VA and NVA	Visual search for static facial expressions	x	x	x	x	x	x	
Mathewson et al. (2011)	ASD; TD	15; 16	CA; FSA	Emotional Stroop (name color of static face stimuli) and identify emotional expression of static faces	x			x			
Rutherford et al. (2012)	ASD; TD	19; 10	CA; FSA	Examination of expression adaptation effects (the phenomenon whereby prolonged exposure to a particular emotion biases one to perceive an opposing emotion on a subsequent neutral face)	x	x	x	x	x	x	
Heaton et al. (2012)	ASD; TD	20; 20	CA; VA; NVA; FSA	Identify emotions in prosodic vocalizations	x	x	x	x	x	x	
Wong et al. (2012)	ASD; social phobia; TD	19; 17; 21	≠CA; gender	Identify static facial expressions of varying intensities	x	x	x	x		x	

*ASD, autism spectrum disorder; HFA, high functioning autism; TD, typically developing; DD, developmental delay; CA, chronological age; VA, verbal ability; NVA, non-verbal ability; FSA, full-scale ability; ≠, groups are not matched on this variable*.

**Table 2:** **Studies examining the brain correlates of emotion perception in ASD**.

Reference	Participant groups	*N*	Matching	Paradigm	Emotional expressions studied							Principal brain related findings
					Happy	Sad	Anger	Fear	Surprise	Disgust	Other	
Critchley et al. (2000)	ASD; TD	9; 9	CA; FSA	Identify expression vs. identify gender of briefly presented expressive faces	x		x					↓lSTG, lLG, ↓rFG, ↓lAmyg (only in implicit gender disk)
Hall et al. (2003)	ASD; TD	8; 8	CA; NVA	Match facial expression with emotional prosody	x	x	x		x			↓lIFG, ↓lLG, ↑rTP, ↑lACC, ↓rFG
Hubl et al. (2003)	ASD; TD	7; 7	CA; NVA	Detect happy faces vs. detect female faces vs. scrambled face baseline vs. geometric shape visual perception task	x	x	x					↓FG and subtle ↓INS
Oagi et al. (2003)	ASD; TD	5; 5	CA; VA; NVA; FSA	Concentrate on identifying static facial expressions (actual performance tested after scanning)	x			x		x		↓lINS, ↓lIFG, ↓lMFG, ↓lputamen
Piggot et al. (2004)	ASD; TD	14; 10	CA; FSA; VA; NVA	Match static facial expressions with one another vs. identify static facial expressions vs. match geometric shapes with one another			x	x	x			↓FG during matching
Wang et al. (2004)	ASD; TD	12; 12	CA; VA	Match static facial expressions with one another vs. identify static facial expressions vs. match geometric shapes with one another			x	x				↓FG, ↓task modulation of rAmyg; marginal ↑precuneus
Dawson et al. (2004)	ASD; TD	29; 22	CA; subgroup also on FSA	Passive viewing of static facial expressions				x				↓Emotion modulation of N300 and NSW (≈800–1200 ms)
Dalton et al. (2005, Exp1.)	ASD; TD	11; 12	CA	Identify whether or not an emotion is expressed in faces with direct or averted gaze	x		x	x				↓FG, ↑lAmyg, ↓rMFG, ↑lOFC
Kujala et al. (2005)	ASD; TD	8; 8	CA	Determine when the prosody of a spoken word (“Saara”) deviated from neutral (on 21% of trials)		x	x				x	↓N300 to anger, topographical differences over frontal electrodes
O’Connor et al. (2005)	ASD; TD	30; 30	CA	Identify static facial expressions	x	x	x	x				Delayed P1, ↓N170; diff. more marked in adults than children
Dapretto et al. (2006)	ASD; TD	10; 10	CA; FSA	Imitate vs. observe static facial expressions vs. null events baseline	x	x	x	x				↓rIFG, ↓rINS, ↓rAmyg, ↑visual processing areas
Ashwin et al. (2007)	ASD; TD	13; 13	CA; FSA	Respond as quickly as possible when a face is shown; faces expressing no fear, mild fear, or extreme fear presented randomly among scrambled faces				x				↓lOFC, ↓lAmyg, ↑rACC, ↑rSTS, ↑STG; ↓signal mod. by fear intensity
Deeley et al. (2007)	ASD; TD	18; 9	CA; FSA; VA; ≠NVA	Identify the gender of static faces expressing various intensities of emotions	x	x		x		x		↓FG, ↓LG, ↓mOG, ↓IOG; ↓signal mod. by intensity
Pelphrey et al. (2007)	ASD; TD	8; 8	CA; VA; NVA; FSA	Respond when a face appears on the screen; static expressive and neutral faces and dynamic emotion and identity morphs were presented			x	x				↓lFG, ↓lMFG, ↓rAmyg, ↓rSFG, ↓MTG, ↓rSTS; ↓mod. by static vs. dynamic emotions
Korpilahti et al. (2007)	ASD; TD	14; 13	CA	Passive listening (while watching a silent cartoon) of two one-word prosodic utterances			x					Ealiere and ↓N1, ↑eMMN (at around 200 ms)
Wicker et al. (2008)	ASD; TD	12; 14	CA	Identify emotion vs. identify age (young/old) of face stimuli where gaze direction changes dynamically from averted to averted or averted to direct	x		x					↓rTPJ, ↓rIFG, ↓SFG; ↓Amyg – frontal and frontal – STS connectivity
Wong et al. (2008)	ASD; TD	10; 12	CA; NVA	Identify gender of or discriminate emotion (against neutral) of static face stimuli	x	x	x	x				No group diff. for P1 and N170; ↑P2 mod. by expression; dipole source diff. in FG, MFG, left cuneus, and precuneus
Corbett et al. (2009)	ASD; TD	12; 15	CA; ≠FSA	Determine whether two static face images are of the same emotion or person vs. whether two static abstract shapes are the same	x	x	x	x				↓rFG, ↓lAmyg, ↑lSPL, ↑lMFG/IFG
Hadjikhani et al. (2009)	ASD; TD	12; 7	CA	Match static body postures of different individuals with one another		x	x	x				↓Amyg, ↓FG. ↓INS, ↓IFG, ↓putamen, ↓premotor, ↓pulvinar, ↓colliculus, ↓accumbens
Grézes et al. (2009)	ASD; TD	12; 12	CA; FSA	Oddball paradigm in which participants needed to detect 10 upside-down stimuli amongst static and dynamic body postures/movements				x				↓rAmyg, ↓IFG. ↓rPrecentral gyrus, ↓rITG (in fear vs. neutral contrast)
Greimel et al. (2010)	ASD; TD	15; 15	CA; VA; FSA; ≠NVA	Identify static facial expressions varying in intensity vs. report own emotional response to the same facial expressions	x	x						↓lIFG, ↓rFG (in self-report condition)
Kleinhans et al. (2010)	ASD; TD	29; 25	CA; VA; NVA; FSA	Match static facial expressions with one another vs. match shapes with one another			x	x				↓lIFG, ↑occipital lobe; rFG, lTP, and rAmyg activity to emotional faces correlated with anxiety
Monk et al. (2010)	ASD; TD	12; 12	CA; VA; NVA	Indicate the left/right position of an asterisk that is shown after static face pairs that include an emotional and neutral expression (or two neutral)	x	x	x					↑rAmyg; ↑rAmyg – ACC, ↓rAmyg – lMTG, and ↓rAmyg – IFG connectivity
Schulte-Rüther et al. (2011)	ASD; TD	18; 18	CA; VA; NVA (marginal); FSA	Identify static emotional expressions vs. report own emotions in response to these expressions								↑rdMPFC, ↓vMPFC, ↓precuneus/PCC in “other” task; ↑rdMPFC, ↑rMFG, ↑rIFC, ↑STS in “self” task
Bastiaansen et al. (2011)	ASD; TD	21; 21	CA; FSA	Observe dynamic facial expressions vs. express emotion vs. experience emotion	x					x		IFG activity correlated with CA only in ASD group
Davis et al. (2011)	ASD; TD	16; 16	CA; VA; NVA; FSA	Passive viewing of static facial expression with averted or direct gaze	x		x	x				↓vlPFC
Weng et al. (2011)	ASD; TD	22; 22	CA; VA; ≠NVA	Identify the gender of static faces expressing emotions; outside scanner identify the emotional expressions	x	x		x				↑Amyg, ↑striatum, ↑IFG; negative Amyg – CA correlation in ASD only

*ASD, autism spectrum disorder; TD, typically developing; CA, chronological age; VA, verbal ability; NVA, non-verbal ability; FSA, full-scale ability; ≠, groups are not matched on this variable. l, left hemisphere; r, right hemisphere; ↑, increased signal contrast in ASD vs. comparison group; ↓, decreased signal contrast in ASD vs. comparison group. ACC, anteriro cingulate cortex; Amyg, amygdala; FG, fusiform gyrus; IFG, inferior frontal gyrus; INS, insula; LG, lingual gyrus; MFG, middle frontal gyrus; dMPFC, dorsomedial prefrongal cortex; vMPFC, ventromedial prefrontal cortex; MTG, middle temporal gyrus; OFC, orbitofrontal cortex; PCC, posterior cingulate cortex; SFG, superior frontal gyrus; STG, superior temporal gyrus; STS, superior temporal sulcus; SPL, superior parietal lobe; TP, temporal pole; TPJ, temporal-parietal junction*.

Although studies of emotion perception are by far the most numerous, there is also a considerable literature on other facets of emotion related interpersonal behaviors in ASD (see Begeer et al., 2008 for a recent review). For instance, it is fairly consistently reported that autistic individuals are less likely than non-autistic individuals to direct emotional expressions at others during naturalistic interactions (Snow et al., 1987; Macdonald et al., 1989; Mundy and Sigman, 1989; Yirmiya et al., 1989; Dawson et al., 1990; Kasari et al., 1990, 1993; Sigman et al., 1992; Dissanayake et al., 1996; Charman et al., 1997; Joseph and Tager-Flusberg, 1997; Bieberich and Morgan, 1998; Zwaigenbaum et al., 2005; Hobson et al., 2009; Hudenko et al., 2009). Autistic individuals also tend to mimic the facial expressions of others less frequently and consistently than non-autistic individuals (McIntosh et al., 2006; Beall et al., 2008; Stel et al., 2008; Oberman et al., 2009; but, see Magnée et al., 2007; see also Sims et al., 2012) and they share the emotional experiences of others in a qualitatively different manner than comparison groups (Yirmiya et al., 1992; Baron-Cohen and Wheelwright, 2004; Lombardo et al., 2007; Rogers et al., 2007; Dziobek et al., 2008; Hobson et al., 2009; Hurdy and Slaughter, 2009; Minio-Paluello et al., 2009a; Bird et al., 2010; Greimel et al., 2010; Schulte-Rüther et al., 2011; Schwenck et al., 2011; see also the discussion between Minio-Paluello et al., 2009b; Smith, 2009). Although findings in this context are not always consistent (see Begeer et al., 2008), the weight of the evidence overall leaves little doubt that ASD is characterized by anomalies in multiple facets of interpersonal affective behaviors, and the real life consequences of these have been documented in a series of elegant naturalistic observations by Sigman and Kasari and their colleagues (Sigman et al., 1992; Dissanayake et al., 1996; Corona et al., 1998; see also Loveland and Tunali, 1991; Bacon et al., 1998; see also Hobson et al., 2009). In these studies, children with and without a diagnosis of ASD were videoed as they interacted with an experimenter or parent who suddenly expressed distress, fear, or discomfort. Not surprisingly, children with learning difficulties and typically developing children responded to the adult’s expressions by interrupting their play behavior and orienting to the adult, often with marked concern. Autistic children, on the other hand, were more inclined to keep playing with their toys even when physiological arousal responses indicated that they had registered the event at some level (Bacon et al., 1998; Corona et al., 1998). An important consequence of this was that autistic children did not acquire avoidance behaviors toward the stimuli that had elicited the negative emotions in the adult. In other words, they were not only less attentive to the emotional displays of others but also missed opportunities to learn about the hedonic significance of objects in their environment as a result.

The evidence set out thus far suggests that autistic individuals are limited in multiple aspects of emotionally patterned communication. Laboratory experiments and naturalistic observations converge in showing that emotional expressions are not particularly salient to autistic individuals and although they identify emotional expressions during some circumstances, they appear not to engage the same processes in order to do so. They also make fewer attempts to initiate emotional exchanges and together these differences afford autistic individuals fewer opportunities to share their emotional experiences with others and to learn about the hedonic significance of environmental stimuli through them (see also Hobson, 1993, 2002). That these disturbances exist and that they have consequences for the developmental trajectory of the disorder is no longer disputed. What continues to divide opinion is what causes this facet of ASD in the first place.

### The theories

Many theoretical frameworks are relevant to the social-emotional characteristics of ASD, including those that identify anomalies in domain-general processes such as perception, attention, learning, and executive function as critically important in mapping the developmental trajectory of the disorder. The focus of this review, however, lies with a group of theories that consider social-emotional difficulties in ASD to be the result of atypicalities in relatively domain specific processes that operate primarily (or even exclusively) in the context social interactions^3^. These theories include the idea that atypicalities in the development of a neural network mediating social-motivation are responsible (e.g., Dawson and Lewy, 1989; Schultz et al., 2003; Dawson et al., 2005; Schultz, 2005; Chevallier et al., 2012), the suggestion that differences in the self regulation of behavior play a critical role (e.g., Loveland, 2005; Bachevalier and Loveland, 2006), the view that a deficient mechanism for the understanding of mental states is to blame (e.g., Leslie and Frith, 1990; Baron-Cohen, 1995; Baron-Cohen et al., 2000; Frith, 2003) and the notion that a disruption in an infant’s readiness to *relate to* and *identify with* the psychological orientations of others lies at the root of the problem (e.g., Hobson, 1993; Hobson, 2002; see also Loveland, 2005).

#### Social-motivational accounts

Social-motivational theories originate in observations of atypical face processing in ASD (see Weigelt et al., 2012 for a critical review). As noted above, one of the early studies by Hobson et al. (1988b) suggested that autistic children engage unusual perceptual processes to extract emotional expressions from faces, which several subsequent studies have confirmed (e.g., Tantam et al., 1989; Davis et al., 1994; Teunisse and de Gelder, 2001; Gross, 2005; Deruelle et al., 2008b). It has also become apparent, however, that atypical face processing interferes not only with the perception of emotional expressions but also with the perception of non-emotional information such as an individual’s identity (e.g., Boucher and Lewis, 1992; Davis et al., 1994; Boucher et al., 1998; Joseph and Tanaka, 2003; Deruelle et al., 2008b; Wallace et al., 2008), age (Hobson, 1983; Gross, 2002, 2005), or gender (Deruelle et al., 2004). Moreover, eye-tracking studies suggest that autistic individuals fixate less on faces than non-autistic individuals when viewing complex social scenes and even when they do, they look less at the eye-region and more at the mouth or non-feature regions of the face (Klin et al., 2002; Pelphrey et al., 2002; Dalton et al., 2005; Speer et al., 2007; Spezio et al., 2007; Corden et al., 2008b; Bird et al., 2011; see Senju and Johnson, 2009 for further discussion). Broader than emotion related atypicalities in social perception are also evident in relation to biological motion (e.g., Moore et al., 1997; Blake et al., 2003; Cook et al., 2009; Koldewyn et al., 2011; Annaz et al., 2012) and vocalizations (McCann et al., 2007; Peppé et al., 2007). Thus, difficulties in the processing of emotional expressions can be seen to constitute only part of a broader anomaly in the perception and understanding of the social environment (i.e., other people).

To explain widespread social-perceptual difficulties, several authors have contributed to a social-motivational theory, which argues that a lack of motivation to attend to and interact with others early in life leads to the divergent development of a social brain network that is critical for the perception and understanding of the social environment (Grelotti et al., 2002; Schultz et al., 2003; Dawson et al., 2005; Schultz, 2005; Chevallier et al., 2012). The basic tenets of this account are the following. First, that human interaction is inherently rewarding. Second, that ASD is the result of a dysfunctional neural network comprising the amygdala, striatum, and orbital-frontal cortex that mediates both the experience and seeking of such reward. Third, that the consequence of this dysfunction is that autistic infants orient less to the social environment, thus compromising the maturation of a broader social brain network that encompasses areas critical for face processing (e.g., Fusiform Gyrus; see Kanwisher, 2000), mental state understanding (Superior Temporal Sulci, Medial Prefrontal Cortex, Temporal Poles, see Gallagher and Frith, 2003), and empathy/interpersonal affective behaviors (Anterior Cingulate Cortex, Anterior Insula, Amygdala, Striatum; see Baron-Cohen, 2005; Singer, 2006; Singer and Lamm, 2009). And finally, social-motivational accounts argue that it is the dysfunction of this broader social brain network that ultimately yields the clinically defining reciprocal social impairments of ASD, including the difficulties we, see in social-emotional behaviors. This account is consistent with a large body of evidence (Schultz, 2005; see Dawson et al., 2005; Grossmann and Johnson, 2007; Chevallier et al., 2011 for relevant reviews) that I will return to in more detail shortly when considering the concept of a “social brain” more closely.

#### The behavioral self regulation account

The view put forward by Loveland and colleagues (Loveland, 2001, 2005; Bachevalier and Loveland, 2006) in many ways complements the social-motivational view. Loveland points out that ASD is characterized by difficulties not only with respect to the perception of social-emotional signals but also with regards to the regulation of behavior in response to these signals. At first, this may seem a trivial point since it should come as no surprise that an individual who experiences difficulties in perceiving certain properties of the world should also respond to these properties differently. Loveland’s arguments, however, are far from trivial, because they stress that perception and action are intimately linked (see Merleau-Ponty, 1964; Fogel, 1993 for additional discussion). Perceiving the emotional significance of someone else’s facial and postural expressions is of little use if one does not know how to respond appropriately, and not understanding the behavioral affordances of emotional signals may be reason enough not to attend to them in the first place. Thus social-emotional difficulties in ASD may not arise because of a lack of motivation *per se* but because of a lack of understanding what emotional signals afford. Support for this argument stems from the studies by Sigman and Kasari and colleagues outlined earlier in which autistic children were consistently less responsive to the emotional displays of others (Loveland and Tunali, 1991; Sigman et al., 1992; Yirmiya et al., 1992) despite demonstrating an awareness of the emotional displays in question (Bacon et al., 1998; Corona et al., 1998). The behavioral self regulation view is also in line with studies that demonstrate typical physiological but atypical behavioral separation anxiety in autistic children (Willemsen-Swinkels et al., 2000; Sigman et al., 2003) and more generally it helps to explain why studies of emotion perception tend to yield less consistent behavioral differences between ASD and non-ASD groups than studies examining the ability to use such expressions to regulate interpersonal exchanges. At the neural level, Bachevalier and Loveland (2006) largely agree with the idea that abnormalities in a social brain network are likely to lie at the root of the developmental trajectory of ASD. They particularly emphasize interactions between the orbital-frontal cortex and amygdala as key to understanding the disorder, citing abundant evidence to support the idea that these areas play a critical role in the self regulation of behavior during social-emotional exchanges.

#### The interpersonal relatedness account

A view closely related to the behavioral self regulation account is that developed by Hobson (1993, 2002, 2012) who, like Loveland (2005), emphasizes the need to consider the social-emotional characteristics of ASD within the context of *inter*personal processes that encompass perception as well as action. Hobson, however, goes one step further by arguing that people do not simply inter*act*, they *identify with* and *share* the psychological orientations and attitudes (including emotional) of others. This concept of “*interpersonal engagement*” may seem difficult to operationalize at first, but observers can reliably determine whether or not two individuals are engaged or not (Hobson and Lee, 1998; García-Pérez et al., 2007). Moreover, very persuasive arguments have been made about the utility of this concept in understanding both typical and atypical forms of interpersonal behavior (Hobson, 1993, 2002, 2012; Hobson and Lee, 1999; Agnetta and Rochat, 2004; Hobson and Meyer, 2005; Meltzoff, 2007; Hobson et al., 2009).

In relation to the social-emotional characterization of ASD, two aspects of Hobson’s theory are important to highlight. First, he stresses that the earliest interactions between an infant and her caretakers are emotionally very rich. Caretakers exaggerate their emotional expressiveness and infants react to these with emotional expressions of their own (see Nadel and Muir, 2005 for a collection of reviews). Second, and similar to Loveland (2001, 2005), Hobson (2002) highlights the fact that perception and action are closely interlinked, which is evident soon after birth in the form of “entrainment” whereby infants synchronize their general motility levels with the patterning of adult speech (e.g., Condon, 1979; Kato et al., 1983). Whether innately specified or not, Hobson argues that the synchronized and emotionally patterned quality of early social exchanges forms an ideal and necessary basis for the development of interpersonal engagement (see also Trevarthen’s concept of “primary intersubjectivity”; e.g., Trevarthen, 1979). It allows the infant first to discover a connection between her own behaviors and that of others, and through that a connection between behaviors and subjective experiences. In other words, the co-ordinated and affectively rich interactions with others early in life, lays the foundation for the infant to discover that other people are “like me.” The argument in relation to ASD is that this capacity to *identify* with others never fully matures. Although Hobson does not emphasize the neural basis of interpersonal engagement and identification, his account is clearly in line with the notion of a social brain dysfunction and resonates with reports of atypical brain correlates of empathy in ASD (Greimel et al., 2010; Schulte-Rüther et al., 2011; but, see Bird et al., 2010)^4^.

#### The mentalizing account

The final explanation to set out before drawing this section to a close is the idea that social-emotional difficulties in ASD are the result of a mentalizing impairment. Mentalizing^5^ refers to the ability to understand, describe, and explain behavior in terms of mental phenomena (e.g., beliefs, desires, intentions, etc…) and it is well established that autistic individuals experience difficulties in this domain across a wide range of contexts (Frith, 2001, 2003; see Baron-Cohen, 2001; Boucher, 2012 for reviews). Given that emotions are, at least in part, mental phenomena, it is relatively straightforward to see how an impairment in mentalizing would have repercussions for emotion related social behaviors. Nevertheless, it is useful to consider one of the most detailed formulations of such an account more closely.

Baron-Cohen (1995, 2005) argues that the ability to mentalize reflects the operation of a Mindreading System that consists of six neuro-cognitive mechanisms – The Intentionality Detector (ID), Eye Direction Detector (EDD), Emotion Detector (TED), Shared Attention Mechanism (SAM), Theory of Mind Mechanism (ToMM), and The Empathizing System (TESS). In typical development, ID, EDD, and TED functionally mature first (between birth and 9 months of age) and endow infants with the ability to comprehend *Agent-Object* relations in terms of mental processes such as “wanting” something (ID), “seeing” something (EDD), or being “angry” about something (TED). In ASD, the functions of ID, EDD, and TED are thought to be qualitatively preserved although their development may be delayed. Next to mature in typical development (between 9 and 18 months) is SAM, which uses the dyadic representations from ID, EDD, and TED to compute more complex triadic representations of *Self-Agent-Object* relations. These representations allow infants to understand that the object of their own mental scrutiny may also be the object of another agent’s mental scrutiny thus marking the beginnings of joint attention behaviors such as gaze monitoring, protodeclarative pointing, and social referencing. The relative absence of such behaviors is the first reliable clinical marker of ASD (Luyster et al., 2009; see Bruinsma et al., 2004 for a review). Since SAM integrates information from EDD and TED, a developmental failure of SAM should result in particular difficulties to extract mental states including emotions from the eye-region of the face. Both eye-tracking (e.g., Dalton et al., 2005) and behavioral evidence (Baron-Cohen et al., 1997, 2001) lend support to this notion. The final components of the Mindreading System to mature are ToMM (between 18 and 48 months) and TESS (around 14 months), which allow the developing child to understand that mental phenomena do not always represent the world as it truly is (ToMM) and to respond to a subset of mental phenomena – emotions – with appropriate empathy (TESS). Both of these competences are compromised in ASD (see Frith, 2003; Minio-Paluello et al., 2009b; Smith, 2009) although arguments have been levied against this conclusion (e.g., Bowler et al., 2005; Bird et al., 2010).

Similar to the accounts set out earlier, the notion of mentalizing difficulties is fully compatible with the idea that ASD is the result of a social brain dysfunction. Superior temporal and medial prefrontal regions involved in mentalizing constitute a core component of the social brain and Baron-Cohen (1995, 2005) highlights the amygdala as particularly important because of its known sensitivity to social-emotional information (Baron-Cohen et al., 2000; Buchanan et al., 2009). At the cognitive-developmental level, the mentalizing account diverges somewhat from the other three theories set out above, which consider the developmental trajectory of ASD to begin at birth, or soon thereafter. The mentalizing framework, by contrast, considers the failure of SAM to develop at 9–18 months as the starting point of the disorder (see Boucher, 2012 for an excellent discussion of the developmental plausibility of mentalizing accounts).

### Summary

I hope to have substantiated the three claims that I set out at the beginning of this section. First, that it is well established that autistic individuals experience difficulties in multiple aspects of interpersonal emotional behaviors and processes. Second, that a group of influential explanations of these limitations consider them as a facet or consequence of broader impairments in reciprocal social competences. And third, that it is widely believed that the neural basis of these impairments is a dysfunctional social brain network comprising regions of temporal and frontal cortex as well as sub-cortical limbic and striatal regions. In the next section I will consider the notion of a “social brain” more closely and suggest that its conceptualization in these terms – i.e., as a *social* brain – ignores many of the functions of its components that are critical for far more domain-general emotion related processes. More specifically, I am going to argue that it is premature to assume that only the “social” functions of the autistic brain are compromised.

## The Social Brain and Its Domain-General Responsibilities

Although the neuropathology of ASD is widespread and characterized by a complex developmental trajectory (Akshoomoff et al., 2002; Redcay and Courchesne, 2005; Courchesne et al., 2011), post-mortem, and structural imaging studies highlight the cerebellum, limbic regions (amygdala, hippocampus, insula, and cingulate cortex), temporal cortical areas (particularly the Fusiform Gyrus and Superior Temporal Sulcus), the dorsal striatum (Putamen and Caudate), and portions of the frontal lobes (Inferior, Medial, Middle, and Superior frontal gyri) as key regions of abnormality in the disorder (see Stanfield et al., 2008; Radua et al., 2010; Cauda et al., 2011; Duerden et al., 2012; Nickl-Jockschat et al., 2012 for reviews). At the functional level, the significance of cerebellar abnormalities is only just beginning to be understood (Strick et al., 2009; Schmahmann, 2010; Halloran et al., 2012) but it is consistently demonstrated that abnormalities in the striatum, limbic system (amygdala and cingulate cortex), temporal cortices (superior temporal sulcus and gyrus, fusiform gyrus, and temporal poles), and frontal cortical areas (medial prefrontal, orbitofrontal, and insular cortices) are linked to facets of the social-emotional impairments characterizing ASD (see Di Martino et al., 2009; Minshew and Keller, 2010; Sugranyes et al., 2011; Philip et al., 2010, 2012; Vissers et al., 2012 for reviews; see also Table 2). An abundance of evidence from neurotypical and other clinical populations furthermore supports a role of these same regions in social cognition and behavior (see Kanwisher, 2000; Gallagher and Frith, 2003; Singer, 2006; Adolphs, 2009; Singer and Lamm, 2009). In short, there is little doubt that “social functions” of the autistic brain operate differently.

A developmental perspective adds further weight to the above conclusion, particularly as formulated by social-motivational theories. The seminal work by Johnson and colleagues demonstrates that social brain functions are not entirely innately specified but rather subject to developmental maturation (see Karmiloff-Smith, 1998; Johnson, 2000, 2003, 2011; Johnson et al., 2009). What does seem to be innately specified is a drive to attend particularly to the social environment, or to stimulus patterns that bring about attention biases to the social world. Soon after birth, for instance, babies preferentially orient to stimulus configurations that contain more elements in the top than the bottom half, thus leading to a preference to attend to face stimuli over most non-face stimuli (Johnson et al., 1991; Valenza et al., 1996; Macchi Cassia et al., 2004). Similarly, they prefer to listen to speech sounds over non-speech sounds (Vouloumanos and Werker, 2004). These early attention biases provide the system with the necessary experience to drive the maturation of more and more specialized social-perceptual and social-cognitive functions. Recent studies suggest that 14 month old toddlers who go on to develop ASD demonstrate less of a preference to orient toward social over non-social stimuli (moving children vs. animated geometric patterns) than toddlers who do not develop the disorder (Pierce et al., 2011). Similarly, toddlers who go on to receive a diagnosis demonstrate atypical brain responses to speech stimuli over non-speech stimuli when they are asleep (Eyler et al., 2012). Thus, the existing evidence clearly supports the notion that the developmental trajectory of ASD is characterized by early emerging anomalies in social orienting and the processing of the social environment more generally. Does this mean that such anomalies are the *cause* of the developmental trajectory of the disorder? Not necessarily. To date, there is no convincing evidence to suggest that autistic infants process *specifically* “social” information differently. It is equally possible that ASD is characterized by more general atypicalities in orienting to and processing of biologically relevant stimuli of which other people are merely an example. A closer look at the concept of the “social brain” will show that this alternative is not farfetched.

History has taught us that describing a collection of neural structures under an umbrella that implies a domain specific function can introduce unwanted biases in our perceptions of what certain parts of the brain are important for. Thus when Papez (1937) first described the neuroanatomical basis of emotions and MacLean (1949) later introduced the concept of the “visceral brain,” they inadvertently diverted attention from the critical role of the hippocampus in memory. In this vein, it is important not to lose sight of the fact that the areas purported to constitute the social brain are involved in a lot more than mediating social behaviors and cognitions (see Adolphs, 2003, 2009). The medial prefrontal cortex, for instance, is not only involved in mentalizing (Gallagher and Frith, 2003) but also more generally in the control of decision making and decision outcome monitoring (Ridderinkhof et al., 2004). The Fusiform Gyrus, though critical for face processing (see Kanwisher, 2000), is also involved in the processing of stimuli relevant to ones’ area of expertise (Gauthier et al., 2000). Middle and superior temporal cortices play a role in language processing (Price, 2010) and social perception (Allison et al., 2000) but also in the general representation of abstract meaning (Binder and Desai, 2011). And of most interest in relation to the social-emotional characteristics of ASD, the striatal, and limbic areas of the social brain are critical for mediating the domain-general interactions between emotion and cognition that are of interest in the series of articles comprising this “Special Topic.”

The amygdala is well known to be critical for alerting us to biologically relevant events in the environment and to prepare us for action by mobilizing physical as well as cognitive resources. Direct sub-cortical afferents from all sensory modalities allow for the “quick-and-dirty” detection of potentially significant stimuli in the environment. Should a rapid response be required, direct efferent connections with hypothalamic and brain-stem nuclei activate arousal systems and “fight-or-flight” responses. A vast network of cortical inputs moderates this sub-cortical system and provides the means for a more controlled response to the environment and a multitude of reciprocal connections with cortical as well as sub-cortical regions allows the amygdala to moderate a wide range of cognitive processes, ranging from perception and attention, through memory and decision making to the most human of capacities – self aware thought (see LeDoux, 1995, 1996; Aggleton, 2000; Lane and Nadel, 2000; Davis and Whalen, 2001; Zald, 2003; Phelps, 2006; Whalen and Phelps, 2009; Dolcos et al., 2011; Ray and Zald, 2012 for a collection of relevant reviews). The dorsal striatum has equally widespread influences. In conjunction with frontal cortical regions and also the amygdala, it is involved in alerting us to reward contingencies in the environment and to facilitates decision making processes to optimize our chances of benefiting from them (e.g., Balleine et al., 2007; Delgado, 2007). There is no doubt that all of these processes are important for navigating the social world successfully – few processes are not! But before we conclude that *only* the processes relevant to dealing with the social environment are compromised in ASD we must examine these broader functions of the “social brain” carefully and consider how they might contribute to the developmental trajectory of the disorder. Closer scrutiny of the mechanisms that mediate the impact of emotion on cognition, in this context, seems particularly pertinent.

## What Do We Know about the Impact of Emotion on Cognition in ASD?

### Arousal responses in ASD

Before we consider how the emotional salience of stimuli impacts on cognitive processes in ASD, it is important to establish to what extent environmental events elicit emotional experiences in this disorder in the first place. Stimulus elicited arousal responses such as changes in cardiac activity, galvanic skin responses (GSRs), or pupil dilation are of considerable interest in this context. Table 3 summarizes studies that have examined such responses in ASD since 1980^6^, grouped according to whether arousal was assessed in response to simple sensory stimuli, stimuli varying in emotional significance, stimuli varying on some social dimension (e.g., emotional expression, absence/presence of significant other, gaze direction, etc.), or stress induction procedures. The results are tabulated in terms of whether groups differed with respect to the overall magnitude of arousal responses (Mag.) and/or the extent to which arousal responses differentiated between stimulus categories and/or experimental conditions (Diff.).

**Table 3:** **Studies examining peripheral arousal responses in ASD**.

Reference	Participant groups	*N*	Matching	Stimuli/paradigm	DVs	Results	
						mag.	diff.
**STUDIES EXAMINING RESPONSES TO SIMPLE SENSORY STIMULI**							
Stevens and Gruzelier (1984)	ASD; TD; DD	20; 20; 20	CA; NVA	Tones of different amplitude	GSR	=	↓
James and Barry (1984)	ASD; TD; DD	40; 40; 40	CA; FSA	Tone vs. white squares	CA; GSR; RP	↑	↓
van Engeland (1984)	ASD; TD; DD; psychiatric	35; 45; 20; 38	CA;	Tone	GSR	↑	=
Barry and James (1988)	ASD; TD; DD	32; 32; 32	CA; FSA	Tones of different amplitudes; white squares of different sizes	CA; GSR; RP	=/↑	↓
van Engeland et al. (1991)	ASD; TD; psychiatric	20; 20; 40	CA; NVA	Meaningless black-white patterns of different complexity; target detection paradigm	GSR	↓	=
Schoen et al. (2009)	ASD; SMD*; TD	38; 31; 33	Not specified	Tone, flash, siren, smell, touch, chair tip	GSR	↓Smell and touch	
Bernier et al. (2005)	ASD; TD	14; 14	CA; FSA; anxiety	Simple fear conditioning paradigm	EMG	=	
Gaigg and Bowler (2007)	ASD; TD	14; 14	CA; VA; NVA; FSA	Differential fear conditioning	GSR	↓	↓
South et al. (2011)	ASD; TD	36; 36	CA; VA; NVA; FSA	Simple fear conditioning paradigm	GSR	=	=
						Correlation: ↑fear acquisition/↓ADOS	
**STUDIES EXAMINING RESPONSES TO EMOTIONALLY SALIENT STIMULI/SITUATIONS**							
Ben Shalom et al. (2003)	ASD; TD	10; 10	CA; ≠VA; NVA; FSA	Rate pleasant, unpleasant, and neutral photographs	GSR	=	=
Salmond et al. (2003)	ASD; TD	14; 18	CA	Startle response modulation by emotional photographs	EMG	=	=
Ben Shalom et al. (2006)	ASD; TD	10; 10	CA	Rate pleasant, unpleasant, and neutral photographs	GSR	=	=
Johnson et al. (2006)	ASD; TD	15; 14	CA; FSA, VA, NVA	Iowa Gambeling Task	GSR	↓	NA
De Martino et al. (2008)	ASD; TD	14; 15	CA; FSA, VA, NVA	Decide whether to choose a certain or uncertain monetary gain/loss	GSR	↑	↓
Bölte et al. (2008)	ASD; TD	10; 10	CA; NVA	Rate pleasant, unpleasant, and neutral photographs	CA; BP	=	↓
Gaigg and Bowler (2008)	ASD; TD	18; 18	CA; VA; NVA; FSA	Study neutral and emotionally salient words for memory test	GSR	=	=
Wilbarger et al. (2009)	ASD; TD	14; 14	CA; VA	Startle response modulation by emotional photographs	EMG	=	↓
Dichter et al. (2010)	ASD; TD	20; 37	≠CA	Startle response modulation by emotional photographs	EMG	=	↓
Maras et al. (2012)	ASD; TD	19/24; 19/24 in two Exp.	CA; VA; NVA; FSA	Memory test for narrated slide-show or video varying in emotional content	GSR; CA	=	=
**STUDIES EXAMINING RESPONSES TO SOCIAL STIMULI**							
Palkovitz and Wiesenfeld (1980)	ASD; TD	10; 10	CA	Tone vs. speech	CA; GSR	↑	=
Corona et al. (1998)	ASD; DD	22; 22	CA; FSA, VA	Simulated distress of adult	CA	↓	NA
Blair (1999)	ASD; TD; DD	20; 20; 20	CA; VA	Distress, threat, and neutral photographs	GSR	=	=/↓
Willemsen-Swinkels et al. (2000)	ASD; TD; DD	32; 19; 19	CA; FSA, VA, NVA	Separation vs. reunion with parent	CA	=	=/↑
Hirstein et al. (2001)	ASD; TD	37; 25	Not specified	Eye-contact with mother vs. papercup	GSR	↓	↓
Sigman et al. (2003)	ASD; DD	22; 22	CA; FSA, VA	Video of crying vs. playing infant; interaction with mother; separation vs. reunion with mother	CA	=	=/↓
Kylliäinen and Hietanen (2006)	ASD; TD	12; 12	CA; NVA	Detect direct vs. averted gaze	GSR	=	=/↑
Naber et al. (2007)	ASD; DD	11; 9	CA; FSA	Separation vs. reunion with parent	CA; cortisol	Number of autistic symptoms predicted lower cortisol response	
Hubert et al. (2009)	ASD; TD	16; 16	CA	Identify angry vs. happy faces	GSR	↓	↓
Bal et al. (2010)	ASD; TD	17; 38	CA; VA; NVA; FSA	Identify dynamic facial expressions as quickly as possible	CA	↑	NA
Riby et al. (2012)	ASD; WS; TD	12; 13; 25	CA	Watch live or videoed dynamic happy, sad, or neutral facial expressions	GSR	=	↓
**STUDIES EXAMINING RESPONSES TO STRESSORS**							
Jansen et al. (2003)	ASD; DD; TD	10; 10; 12	CA; FSA	5 Min public speaking (social stress test)	CA; cortisol	↓CA	NA
Toichi and Kamio (2003)	ASD; TD	20;20	CA; VA; NVA	Rest vs. mental arithmetic	CA	=	=/↓
Corbett et al. (2006)	ASD; TD	12; 10	CA; ≠FSA	20 Min mock MRI scan	Cortisol	↑	NA
Corbett et al. (2008)	ASD; TD	22; 22	CA; ≠FSA	20 Min mock MRI scan followed by real scan for subgroup	Cortisol	=	=						Higher variability in ASD group	
Corbett et al. (2009)	ASD; TD	22; 22	Not specified but statistically controlled	20 Min mock MRI scan	Cortisol	=	=						Higher variability in ASD group	
Jansen et al. (2006)	ASD; TD	10; 14	CA; VA; NVA; FSA	10 Min public speaking (social stress test)	CA; cortisol; adrenalin	↓CA	NA
Levine et al. (2012)	ASD; TD	19; 11	CA; FSA	10 Min public speaking and other exercises (trier social stress test)	CA; cortisol	↓Cortisol	=

*ASD, autism spectrum disorder; HFA, high functioning autism; TD, typically developing; DD, developmental delay; CA, chronological age; VA, verbal ability; NVA, non-verbal ability; FSA, full; CA as a dependent variable, cardiac activity; GSR, galvanic skin responses; EMG, elecromyogramm; BP, blood pressure; RP, respiratory pause*.

*↓, Decreased response magnitude or differentiation in ASD; ↑, increased response magnitude or differentiation in ASD; =/↑ and =/↓, indicate that responses were atypical in relation to only a subset of stimuli and/or in only a subgroup of participants. NA, not applicable*.

Overall, the literature is clearly extremely varied. Importantly, however, there is little evidence to suggest that autistic individuals are either consistently hyper- or hypo-aroused by the social environment. Rogers and Ozonoff (2005) reached a similar conclusion in relation to simple sensory stimuli and it is curious that both the sensory and social environment should yield a mixed pattern of arousal responses in ASD. This varied pattern contrasts the observations of studies examining arousal responses to emotionally salient pictures, words, or narratives where responses to different stimulus categories are sometimes less differentiated but on the whole response magnitudes are relatively preserved. One could formalize this pattern by suggesting that autistic individuals only demonstrate typical arousal responses in relation to stimuli that are relatively unambiguous and invariable with respect to their emotional significance (e.g., spiders, startling noises, profanities, etc.). Responses to more ambiguous stimuli (e.g., arbitrary sensory stimuli or other people), on the other hand, are compromised in a manner that leads to very variable patterns of arousal across contexts. To illustrate, consider the contrast between the emotional significance of profanities and that of the ever-changing behaviors of other people. Profanities, no doubt, acquire their emotional significance within the context of social interactions. Early in development, they are typically encountered in highly emotive situations. A toddler might utter a profanity not knowing what it meant and be scolded, or he may witness a heated argument and be frightened by the aggression on display. Either way, encounters with profanities would fairly reliably be associated with the experience of fear. By contrast, encounters with other people are associated with a whole range of emotional experiences. One moment the adult smiles, the next he scolds and after that he may look puzzled, indifferent, or surprised. Probabilistically, therefore, profanities are associated much more reliably with a particular pattern of emotional experience than encounters with other people (or ambiguous sensory stimuli) and studies of associative learning suggest that this difference is critical in the context of ASD.

### Learning about the emotional significance of environmental events

One of the most important functions of the amygdala is to mediate the associative learning processes through which an organism is able to predict potential danger or reward. The nature of these processes has been studied most extensively through aversive and appetitive conditioning paradigms in which essentially neutral sensory stimuli come to elicit avoidance or approach behaviors because of their predictive validity over inherently harmful or pleasant events (for detailed reviews, see LeDoux, 1995, 1998, 2002; Murray et al., 2009; Öhman, 2009). Four studies have examined aversive conditioning in ASD to date.

#### Aversive conditioning

Bernier et al. (2005) examined fear conditioning through a fear potential startle paradigm in which participants were presented with consecutive trials comprising a red square (the Conditioned Stimulus, CS) that co-terminated with an overlapping 50 ms aversive air-puff to the throat (the unconditioned Stimulus; UCS). Following these acquisition trials, participants’ eye-blink startle responses were examined to bursts of white noise that were either preceded by the red square (“threat trials”) or not (“safe trials”). The results showed that both autistic and non-autistic individuals exhibited augmented eye-blink startle responses during the threat as compared to the safe trials, suggesting that the red square had acquired aversive properties for both groups. Gaigg and Bowler (2007) examined fear conditioning in a somewhat more complicated paradigm in which participants saw a random sequence of four colors, of which one (CS+) was paired with a startling foghorn sound (UCS) on a random 6 of its 12 presentations. The remaining colors (CS−) were never paired with the UCS and thus served as “safe” trials. Examination of GSR showed that fear acquisition to the CS+ color was significantly attenuated in autistic as compared to non-autistic individuals.

South et al. (2011) have recently employed a paradigm that sits somewhere in between those of Bernier et al. (2005) and Gaigg and Bowler (2007) in terms of the stimulus contingencies. Similar to Gaigg and Bowler (2007) these authors employed a differential conditioning paradigm involving a CS+ color that was paired with a startling noise UCS during acquisition and a CS− color that was never paired with the UCS. Similar to Bernier et al. (2005), however, South and colleagues always paired the CS+ color with the UCS during the acquisition trials whereas in Gaigg and Bowler (2008) the UCS followed the CS+ on only 50% of trials. Across three experiments that employed tones, colors, or angry face photographs as conditioned stimuli, South et al. (2011) observed no decrements in fear acquisition in ASD (GSR served as the dependent measure). Importantly, however, the authors did find that the amplitude of acquired fear responses was associated with autism symptom severity such that greater conditioned responses were associated with lower scores on the ADOS Reciprocal Social Interaction scores (i.e., fewer/less severe difficulties in this domain).

In the most recent fear conditioning report in ASD to date, South et al. (in press) employed a reversal paradigm modeled on Schiller et al. (2008). Here participants were presented with a random sequence of two colors of which one (CS+) co-terminated with an aversive puff of air to the throat (UCS) on 33% of trials. Unannounced, the contingency between color and air-puff reversed such that the previously unpaired CS− color now co-terminated with the UCS whilst the original CS+ color no longer did. The results showed that whilst fear acquisition in the first phase was preserved in ASD, reversal learning was significantly compromised such that autistic individuals were significantly slower to adapt to the new stimulus contingencies.

Importantly, in all four aversive conditioning studies to date, ASD and non-ASD individuals did not differ with respect to their physiological responses to the UCS, which suggests that the emotional salience of these aversive stimuli was similar for both groups. Although further studies are clearly needed to clarify what factors are critical for determining whether or not fear acquisition is preserved in ASD (number of competing stimuli, probability of reinforcement, timing of events, etc.), it seems clear already, that autistic individuals have difficulties adapting to ambiguous stimulus contingencies that would normally allow one to predict the occurrence of biologically relevant events on a probabilistic basis. In other words, ASD seems to be characterized by anomalies in the mechanisms by which emotional salience facilitates associative learning under conditions of uncertainty. Studies of reward contingency learning lend further support to this conclusion.

#### Reward contingency learning

Learning to predict when and where to expect rewards is mediated by a complex neural network involving interactions between many parts of the “social brain” including the amygdala and orbital-frontal cortex (Holland and Gallagher, 2004) and the striatal reward system (Delgado, 2007). Johnson et al. (2006) were the first to examine reward contingency learning in ASD by drawing on a well established decision making paradigm known as the Iowa Gambling Task (IGT; Bechara et al., 1997, 2005). Participants in this task are told to try to make as much money as possible by choosing cards from one of four decks, with each choice yielding a certain monetary gain and loss (e.g., you win $0.50 but also lose $0.75). Two decks yield on average more gains than losses but the distribution of rewards across trials is extremely unpredictable. Johnson et al. (2006) found that autistic individuals were significantly slower to develop choice preferences for the advantageous decks than comparison individuals in this task. Solomon et al. (2011) recently observed similar group differences in a probabilistic learning task in which participants needed to discover which of a pair of symbols had the highest chance of being “correct.” For different pairs of symbols, accurate feedback was given on only 80, 70, or 60% of trials and the learning profiles of ASD participants showed that they were less affected by these varying contingencies than comparison individuals (autistic individuals were less likely to demonstrate a “win-stay” pattern of decisions). Finally, Scott-Van Zeeland et al. (2010) examined reward contingency learning in ASD in the context of a categorization task that required participants to classify abstract fractal-like images as belonging to either one of two categories. Correct classifications were rewarded either with a certain amount of money or with a smiling female face whilst incorrect classifications yielded either no reward or a sad female face. Similar to Johnson et al. (2006) and Solomon et al. (2011), the reward contingencies were not entirely reliable such that on some blocks of trials inaccurate feedback was given. Again, autistic individuals were significantly worse at the categorization task – so much so that, in both the face and monetary reward conditions, their performance remained at chance whilst that of the comparison group reached over 80%.

Several studies, including that by Scott-Van Zeeland et al. (2010), have examined the neural correlates of reward processing in ASD and the overall consensus in this context is that striatal as well as frontal areas involved in predicting and acting upon rewards in the environment are functionally compromised in this disorder. Importantly, neural abnormalities are evident irrespective of whether the reward comes in the form of a social smile, money, or simple praise (Schmitz et al., 2008; Kohls et al., 2011; Dichter et al., 2012a,b)^7^. Together with the studies of fear conditioning, this evidence strongly suggests that it is not only the “social functions” of the “social brain” that are compromised in ASD. Instead, autistic individuals have difficulties adapting to stimuli that predict biologically relevant events only imperfectly, irrespective of whether these stimuli are other people or not. At the neural level this suggests anomalies in how basic associative learning processes mediated by sub-cortical amygdala systems are modulated by “higher-level” perceptual and conceptual processes that are critical for resolving ambiguities in the environment. Evidence from other domains suggests that this may be part of a broader disruption of the interplay between basic emotional response systems (i.e., sub-cortical amygdala circuitry) and cognition.

### The emotional modulation of attention in ASD

Besides eliciting arousal responses and facilitating associative learning, the amygdala also plays a critical role in orienting attention toward emotionally significant stimuli (see Vuilleumier, 2005, 2009). To date, only three studies have examined the emotional modulation of attention in ASD in relation to stimuli other than emotionally expressive others. South et al. (2008) employed a visual search task in which participants were asked to determine whether all images in a 3 × 3 display were of the same category or not. The arrays either comprised images of spiders, snakes, flowers or mushrooms, or one of these (the “odd-one-out”) amongst eight of the others. Both autistic and non-autistic participants demonstrated a well established “threat advantage” effect, whereby the detection of threatening (snakes, spiders) stimuli amongst neutral ones (mushrooms, flowers) was faster than the detection of neutral stimuli amongst threatening ones. Interestingly, autistic individuals have also been shown to demonstrate a typical anger superiority effect in visual search tasks employing face stimuli (Ashwin et al., 2006a; Krysko and Rutherford, 2009; Rosset et al., 2011; but, see Farran et al., 2011)^8^, suggesting that basic threat detection mechanisms may be functionally preserved irrespective of whether the danger comes from the social environment or not. Contrasting these observations from visual search paradigms, Corden et al. (2008b) and Gaigg and Bowler (2009a) observed atypical emotional modulation of the Attentional Blink (AB) phenomenon in ASD. In their studies, participants were presented with rapid sequences of words comprising several distracter items and two target words (T1 and T2) that participants were asked to identify. T1 was always neutral whereas T2 was either neutral or emotionally charged and across trials these two target items were separated by varying temporal delays. Typically, the detection of T2s is significantly attenuated in a period of approximately 100–500 ms following T1 but this “AB” is partially overcome when T2 is emotionally charged (e.g., Keil and Ihssen, 2004). Both Corden et al. (2008b) and Gaigg and Bowler (2009a) found that this emotional modulation of the AB was diminished in ASD.

Further studies are clearly needed to clarify to what extent emotionally salient events capture attention in ASD. In particular it will be important to resolve an apparent paradox between the studies outlined here and the evidence concerning emotional arousal responses in ASD outlined earlier. As noted earlier, arousal responses to stimuli such as emotionally significant pictures or words appear to be relatively preserved in ASD (Ben Shalom et al., 2006; Gaigg and Bowler, 2008) whereas responses to the social environment including the emotional expressions of others are much more varied (e.g., Hubert et al., 2009 vs. Bal et al., 2010). This appears at odds with the studies outlined in this section, which show that facial expressions of certain emotions and images displaying threats capture attention relatively typically in ASD whereas emotionally charged words do not. Importantly, the mechanisms that regulate peripheral arousal responses operate in parallel to those that regulate attention. The former are mediated primarily by sub-cortical connections between the amygdala’s central nucleus and various brain-stem nuclei (e.g., LeDoux, 2002) whereas the latter involve reciprocal connections between the basal/lateral nuclei and sensory processing cortices, and between accessory basal nuclei and the orbital-frontal cortex (see Vuilleumier, 2005, 2009). It is possible, therefore, that sub-cortical amygdala pathways that mediate the expression of physiological arousal responses are relatively preserved in ASD, whilst those that moderate attention through reciprocal connections with cortical areas are compromised. Preserved attention modulation by inherent threat signals such as angry expressions and the sight of a spider could be accommodated within such a view because such stimuli are thought to capture attention by activating relatively simple sub-cortical response systems. The facilitation of attention in response to emotional words, by contrast, is likely to involve more extensive cortico-amygdala networks that include language processing areas.

#### Memory for emotionally salient events in ASD

Another important function of the amygdala is to facilitate declarative memory for emotionally significant events through connections with the hippocampus (see Phelps, 2004; Reisberg and Hertel, 2004; Uttl et al., 2006 for reviews). Six studies have explored this issue in ASD to date. The first was a study by Beversdorf et al. (1998) who presented participants with audio recordings of 10 emotional (e.g., “Carl shot his gun at someone”) or 10 neutral (e.g., “Mike is talking on the phone”) statements. Following each set participants were allowed unlimited time to write down as many statements as possible and results showed that only non-ASD participants recalled the emotional statements better than the neutral ones. Deruelle et al. (2008a) extended these observations to pictorial stimuli using a speeded recognition task. Here participants were asked to study sets of images including six neutral, six positive, and six negative items that were presented for only 150 ms each. Following every set participants were required to discriminate images they had seen from new ones within 1.5 s and in line with Beversdorf et al. (1998) only non-ASD participants demonstrated a memory advantage for emotional over neutral images.

South et al. (2008) and Maras et al. (2012) recently failed to demonstrate quantitative anomalies in memory for emotional over neutral material in ASD. In the two experiments by Maras et al. (2012), participants were presented with a neutral or emotionally charged audio-narrated slide-show (Exp. 1) or video clip (Exp. 2) for which memory was subsequently assessed through free recall and forced choice recognition procedures. In both experiments participants who viewed the emotional versions of the narratives demonstrated superior memory to those who viewed the neutral narratives and this emotional modulation of memory was similar for ASD and non-ASD participants. In the study by South et al. (2008) participants were asked to study a mixed list of negative (high and low arousal), positive (high and low arousal), and neutral words for a subsequent recognition test and here too autistic individuals demonstrated a preserved memory advantage for emotional words. Importantly, however, ASD participants in this study took significantly longer to respond during the test phase, which, in light of the observations of the speeded recognition test by Deruelle et al. (2008a) outlined above, may indicate that qualitative differences in *how* emotion modulates memory in ASD may go undetected by some experimental paradigms – reminiscent of studies of facial emotion recognition in this disorder (see The Evidence).

Two studies by Gaigg and Bowler (2008, 2009b) lend some support to the above conclusion. Gaigg and Bowler (2008) also failed to note group differences in memory for emotional over non-emotional words when comparing the free recall of autistic and non-autistic participants on an immediate test of memory. Importantly, however, the authors included categorically related neutral words (items of fruit) in their study and examined forgetting rates over time by testing free recall again after 1 and 24 h delays. Critically, these features of the experiment revealed that the memory advantage for emotional over non-emotional items on the immediate recall test was commensurate with the advantage for items of fruit over unrelated neutral words, both for ASD and non-ASD participant groups. Over time, however, comparison participants began to demonstrate a specific memory advantage for emotional over non-emotional items whereas autistic individuals did not. In other words, the memory advantage for emotional items observed by South et al. (2008) may simply be a reflection of the effects of semantic factors on memory rather than emotional factors and the narrative structure of the materials used by Maras et al. (2012) may play a similar role. The second study by Gaigg and Bowler (2009b) took a somewhat different approach by focusing on false rather than veridical memory. Participants here were asked to study lists of words comprising orthographic neighbors of critical non-presented items that were either emotionally charged (e.g., tell, fell, sell,… for *hell*) or neutral (e.g., look, took, book,… for *hook*). On a subsequent recognition test non-autistic participants were very unlikely to falsely endorse emotional items as having been studied but ASD participants made such intrusion errors as often for emotional as non-emotional words.

As is the case for studies of attention, further evidence is needed in order to clarify under what circumstances emotion facilitates memory in ASD typically and under what circumstances memory for emotional material may be hindered. Critical, in this context, is to bear in mind that ASD is generally characterized by a particular pattern of memory strengths and weaknesses (see Boucher and Bowler, 2008; Boucher et al., 2012) that may well contribute to inconsistent observations. On balance, however, it does not appear to be the case that emotional factors exert entirely typical effects on long-term declarative memory in ASD.

### The subjective experiences of feelings in ASD

The final issue to consider before drawing this section to a close concerns one of the most elusive questions – how do autistic individuals subjectively experience emotional stimuli? As noted in the preliminary comments, I use the term “feeling” in this review to describe the subjective experiences that arise when the perception of a stimulus or event triggers changes in arousal that, in turn, alter self-conscious thought. Although notions of consciousness and self-awareness continue to be the source of considerable debate, immense progress has been made over the past few years in tracing the neurobiological and psychological origins of subjective experiences. The emerging consensus in this context is that subjective experiences such as feelings critically depend on the convergence of representations of internal body states with representations of the sensory environment in medial frontal cortices and in particular the insula cortex, both of which are under the influence of the amygdala (Damasio, 1994, 1999, 2003; LeDoux, 2002; Critchley et al., 2004; Wiens, 2005; Craig, 2009; Critchley and Nagai, 2012; Seth et al., 2012).

Feelings have recently attracted a lot of attention in ASD because a series of studies have shown that a condition known as Alexithymia is commonly associated with the disorder (Hill et al., 2004; Tani et al., 2004; Berthoz and Hill, 2005; see also Fitzgerald and Bellgrove, 2006; Hill and Berthoz, 2006). Alexithymia is characterized by difficulties in identifying and communicating ones’ own emotions and whilst it affects only approximately 10% of the general population (Mattila et al., 2006), the prevalence in ASD is estimated to be somewhere between 40 and 50% (Hill et al., 2004; Tani et al., 2004). An appealing explanation for this high co-morbidity is that it is a reflection of mentalizing difficulties. In non-autistic individuals, high levels of Alexithymia are associated with poor mentalizing (e.g., Moriguchi et al., 2006) and since autistic individuals are thought to experience difficulties not only with the understanding of other peoples’ minds but also their own minds (see Williams, 2010 for an excellent discussion), high levels of Alexithymia are to be expected. Alternatively, however, high levels of Alexithymia in ASD may be regarded as the result of a disruption in the interplay between emotional arousal and subjective experience. In other words, rather than having difficulties in accessing or reflecting on their feelings, autistic individuals may experience feelings differently because the processes that give rise to them are disrupted. Ben Shalom (2000) was the first to raise this possibility and several studies lend support by showing that physiological arousal responses and subjective ratings of hedonic value in ASD do not co-vary typically (Ben Shalom et al., 2003, 2006; Bölte et al., 2008; Gaigg and Bowler, 2008; Dichter et al., 2010; see also Allen and Heaton, 2010). Moreover, a recent fMRI study has shown that the degree of anterior insula activity during emotional introspection in autistic and non-autistic individuals was moderated by scores on standardized Alexithymia questionnaires whereas activity in the mentalizing system was not (Silani et al., 2008; see also Ebish et al., 2011).

Interestingly, Bird et al. (2010, 2011) have recently shown that self-reported levels of Alexithymia account for abnormalities in empathic brain responses, atypical gaze fixations on the eye-region of faces, and poorer facial emotion recognition in ASD (Cook et al., in press), which suggests that difficulties in introspecting on own emotions and aspects of the reciprocal social impairments in ASD share a common neuro-cognitive basis. The nature and developmental origin of this common denominator remains unclear. One possibility is that ASD *per se* is not at all associated with emotion related anomalies but that the difficulties in this domain are fully explained by the high incidence of Alexithymia. Such a view, however, would leave unexplained why Alexithymia should co-occur with ASD far more often than would be expected by chance. A more appealing account, sees Alexithymia in ASD as a reflection of the same aberrant developmental process that also gives rise to varying social and emotion related difficulties. On this view, one might expect the nature of Alexithymia in ASD to differ qualitatively from that observed in non-autistic individuals and future studies are urgently needed to address this issue. In particular, it will be important to seek objective measures of Alexithymia that go beyond the self-report questionnaires currently available and it will be critical to clarify the developmental trajectory of Alexithymia in autistic as well as non-autistic individuals.

## Reconsidering the Role of Emotion Related Processes in the Development of Autism Spectrum Disorders

I have reviewed evidence demonstrating that ASD is characterized by widespread and pervasive difficulties in social-emotional competences. Autistic individuals often struggle to identify the emotional expressions of others, they frequently do not reciprocate these expressions in a context appropriate manner and they often relate and react to the emotional experiences of others in atypical ways. A group of influential explanations of this facet of the autistic phenotype attribute it to broader abnormalities in social-cognitive processes that find their origin in an early emerging disruption in social-motivation and interpersonal engagement. Put simply the argument is that autistic infants are less motivated (or otherwise hindered) to engage and interact with others, which affords them fewer opportunities to learn from interpersonal experiences and ultimately leads to the clinically defining reciprocal social impairments of the disorder, including anomalies in emotion related interpersonal behaviors. At the neural level, this developmental cascade is thought to reflect abnormalities in the maturation of the “social brain,” which comprises frontal and temporal cortical regions as well as parts of the striatum and limbic system (in particular the amygdala).

In the two sections that followed, I raised concerns about the view that emotion related processing anomalies in ASD need to be understood with reference to broader “social” impairments. I pointed out that many components of the “social brain” are critically involved in far more than the mediation of reciprocal social competences. In particular, the limbic and striatal regions encompassed by the social brain are well known to play a critical role in the domain-general emotion related processes that serve to alert an organism to biologically relevant events in the environment so that it may respond to them effectively and learn about contingencies that are likely to predict similar events in the future. In the light of these domain-general functions, greater empirical effort needs to be directed at establishing whether or not the emotion-related difficulties in ASD are merely a facet of social impairments.

In section three, I summarized the available evidence that speaks to this issue to date. I first considered evidence concerning peripheral arousal responses in ASD, which are widely agreed to constitute an important facet of emotional responses to the environment. Although the evidence in this context is very mixed, I noted that there is little support for the notion that autistic individuals respond specifically to their social environment with atypical patterns of arousal. Instead they appear more generally to react differently to objects and events that are either ambiguous (e.g., arbitrary sensory stimuli) or variable (e.g., other people) with respect to their emotional significance, which contrasts their relatively typical responses to clearly defined and invariable emotional signals (e.g., emotionally significant words, pictures, narratives, etc.). After hinting at the possibility that it is the ambiguous and unpredictable nature of certain events in the environment that poses difficulties for autistic individuals, I summarized evidence from aversive conditioning and reward contingency learning paradigms that is consistent with this suggestion. More specifically, I showed that autistic individuals experience difficulties learning about the (emotional) significance of stimuli that predict biologically relevant events or opportunities only imperfectly. They fail to anticipate danger under ambiguous circumstances and do not adopt efficient decision strategies to take advantage of uncertain reward opportunities. When the environment is regular and predictable, by contrast, they are indistinguishable from non-autistic individuals. Importantly, this pattern holds irrespective of whether or not social signals or arbitrary symbols serve as predictors of relevant events. At the neural level, this suggests that relatively basic amygdala functions that are mediated primarily by sub-cortical networks involving sensory efferents from the thalamus and afferents to brain-stem nuclei are preserved in ASD. Functions that rely on the modulation of this sub-cortical system by other cortical or sub-cortical areas, by contrast, appear to be compromised. I concluded section three with an overview of evidence from studies of attention, memory, and the subjective experience of “feelings” that are all consistent with the notion that interactions between “basic” amygdala networks and the rest of the brain may be compromised in ASD, which, incidentally, is also consistent with evidence of widespread abnormalities in interregional connectivity in the autistic brain (Cheng et al., 2010; Schipul et al., 2011; Shukla et al., 2011; Duerden et al., 2012; Vissers et al., 2012). In short, I outlined evidence that is beginning to lead us to question the notion that anomalies in interpersonal emotional behaviors in ASD are best understood with reference to “social” impairments.

### Implications for developmental accounts of ASD

There is no question that social-motivational accounts of ASD provide an elegant explanation for the impairments in reciprocal social competences that clinically define the disorder. As appealing as such theories are, however, they seem insufficient as explanations for the broader emotion related anomalies I have set out in this review. This may seem relatively unproblematic at first. As Chevallier et al. (2012) point out, failing to explain all facets of the ASD phenotype is “…*only problematic if one considers that there ought to be a single explanation behind all the symptoms of ASD… if one agrees that ASD should be studied from a multiple-deficit perspective,… it is important to compare the explanatory power of social-motivation vs. social cognition in accounting for social deficits*” (p. 236). This argument would be perfectly acceptable, were it not for the evidence set out in this review, which links the social and broader emotion related atypicalities at the neural level. Parsimony, therefore, dictates that we seek a developmental explanation that unifies at least the social and wider emotion related characteristics of ASD and one of the most pressing questions in this context is how best to conceptualize the causal relation between the social-emotional and broader emotion related anomalies set out in this review.

One possibility is that “social” explanations are correct after all and that the broader emotion related impairments reviewed in this paper are a direct consequence of abnormalities in the development of reciprocal social competences. Many of the findings set out in section three are amenable to such a view because it is undoubtedly true that we come to appreciate the emotional significance of objects and events in our environment in the context of rich social interactions (e.g., Bacon et al., 1998; Corona et al., 1998). One may counter this argument by pointing out that it is highly unlikely that atypicalities in the domains of fear conditioning and reward contingency learning would result from abnormalities in reciprocal social development. Cortical areas thought to constitute a critical component of the “social brain” – in particular frontal areas – mature much later in life than the amygdala (e.g., Happaney et al., 2004; Bachevalier and Loveland, 2006), rendering it improbable that the abnormalities that underlie atypical fear conditioning result from atypicalities in the maturation of the “social brain.” Even this argument, however, is far from decisive because the highly complex nature of social interactions may well provide the kind of experience that drives not only the specialization of “social” functions of the brain (e.g., Johnson, 2000, 2003, 2011), but also the maturation of inherently non-social functions such as those involved in certain conditioning paradigms. On this account social interactions could be seen to lead to the development of neural circuitry that is intricately tuned to dealing with rapidly changing, ambiguous, and often unpredictable environments. Perturbations in this process as a result of impoverished experiences with the social environment could thus lead to more widespread abnormalities in how an individual adapts to ambiguous and unpredictable situations.

It is clear from the above discussion, that it would be pre-mature to abandon “social” explanations of ASD altogether. It is equally clear, however, that we should no longer simply accept the assumptions on which such explanations are based. We need to begin to take an alternative seriously, one in which impairments in reciprocal social competences are the result of abnormalities in the domain-general processes that typically allow an infant to rapidly learn about the emotional significance of objects and events in its environment. As I have suggested in section three, the associative processes that mediate such learning may be compromised in ASD such that autistic individuals find it difficult to adapt to the (emotional) significance of particularly those stimuli that predict biologically relevant events only imperfectly. Other people happen to be the most ambiguous and unpredictable “stimulus” of all in this respect as they are not only biologically relevant in their own right but they also signal a whole range of biologically relevant events. One moment a smile indicates the imminent arrival of food, the next the playful withdrawal of a preferred toy and the next it is a simple sign of affection that relates to nothing in the external environment at all. And if this were not complex enough, the same face that produces smiles in a dozen different contexts is also the source of a multitude of other, mutually incompatible emotional signals that occasionally occur in the same context as the smile (e.g., a playful frown whilst withdrawing the preferred toy). Making sense of this organized chaos is a computational nightmare that the typically developing brain appears to master in a matter of months. For autistic individuals, however, the unpredictable nature of the social environment appears to remain to a large extent impenetrable.

## Conclusion and Future Directions

I have attempted to provide a complete overview of what we currently know about emotion related processes in ASD. On balance, the literature suggests that we may need to reconsider one of the core assumptions that underlies most dominant theories concerning the social-emotional difficulties characterizing the disorder, namely that these difficulties are simply a facet of broader social-motivational or social-cognitive impairments. Should the assumption not withstand empirical scrutiny, we face the difficult task of acknowledging that our conceptualization of the developmental trajectory of ASD as originating in impairments in “social” processes may in fact be false. Fortunately, this prospect is not as daunting as it seems, as there are appealing alternatives on the horizon. In particular, Pellicano and Burr (in press), in a very different context, have recently argued that autistic perception is relatively unbiased by prior experience. More specifically, they argue that whereas typical individuals anticipate, rather than experience the world, autistic individuals perceive the world as it actually is. At a neural level, this phenomenological description of the subjective experiences of autistic individuals is anchored in faulty computational mechanisms that iteratively formulate expectations of what might occur next on the basis of experience. These mechanisms are critical for our survival because they allow us to anticipate biologically relevant events, and in the context of our social environment, they allow us to predict the unpredictable. Pellicano and Burr (in press) suggest that ASD is the result of abnormalities in how current experience updates predictions for the future and this seems a promising idea to pursue in relation to the emotion related experiences of autistic individuals.

## Conflict of Interest Statement

The author declares that the research was conducted in the absence of any commercial or financial relationships that could be construed as a potential conflict of interest.

## Footnotes

^1^A draft version of the DSM-V can be found here http://www.dsm5.org/proposedrevisions/pages/proposedrevision.aspx?rid=94

^2^Relevant studies were identified through cited reference searches and through a combination of search terms (“ASD” or “Autism,” “Emotion Perception,” “Emotion Recognition”) in the Web of Knowledge. Studies are only included in the tables if (a) they included a comparison group, (b) the minimum sample size was at least five ASD and five comparison participants, and (c) at least one of six basic emotions (happy, sad, angry, fear, surprise, and disgust) was studied. The last criterion was loosened somewhat to allow for the inclusion of four studies that used other concretely defined emotions of interest (Hillier and Allison, 2002; Peppé et al., 2007; McCann et al., 2007; Shamay-Tsoory, 2008).

^3^Readers interested in the broader theoretical literature relating to ASD should consult the exhaustive overview by Bowler (2007).

^4^This study showed that atypical empathic brain responses in ASD can be accounted for by co-morbid Alexithymia in this disorder, a point to which I will return in more detail in Section “Reconsidering the Role of Emotion Related Processes in the Development of Autism Spectrum Disorders.”

^5^Although the term “Theory of Mind” is often employed to describe this ability, “mentalising” is preferred here to avoid the implication that mental states are understood through a process of theorising or inference – a view that is endorsed by some (e.g., Astington and Gopnik, 1991; Gopnik et al., 1999; Perner, 1991) but not all (e.g., Gordon, 1996; Hobson, 1991, 1993, 2002).

^6^Earlier studies are included in a review of the sensory processing literature by Rogers and Ozonoff (2005) but they are not included here because of the shift in the conceptualisation of ASD as marked by the publication of Wing and Gould (1979). Studies included in Table 3 were identified primarily through cited reference searchers and a Web-of-Knowledge search using the terms arousal and autism.

^7^Scott-Van Zeeland et al. (2010) argue that their data supports the idea that particularly “social” rewards are processed atypically in ASD but they do not provide the relevant Reward Condition × Group analyses, nor do their within condition analyses provide convincing evidence for this conclusion.

^8^Ashwin et al. (2006a), Rosset et al. (2011), and Krysko and Rutherford (2009) all observed relatively typical anger superiority effects but subtle differences between groups in how the number of distracters affected performance. Ashwin et al. (2006a) suggests that these subtler differences are a reflection of more general face processing difficulties and it is worth noting that atypical effects of distracter numbers have also been noted in attention tasks employing letters as stimuli (Remington et al., 2009). Farran et al. (2011) failed to demonstrate an anger superiority effect, observing an advantage for happy expressions instead – this happy advantage was preserved in ASD. Although the ASD group was somewhat slower than TDs to respond to angry, sad and fearful faces this speed decrement was not significant when comparing autistic individuals to verbal ability matched comparison participants.
